# Prevention of cervical cancer in HIV-seropositive women from developing countries: a systematic review protocol

**DOI:** 10.1186/s13643-017-0484-9

**Published:** 2017-04-24

**Authors:** Witness Mapanga, Ahmed Elhakeem, Shingairai A. Feresu, Fresier Maseko, Tsungai Chipato

**Affiliations:** 1 School of Health Systems and Public Health, Epidemiology and Biostatistics, University of Pretoria, 5-10 H.W. Snyman Building, Pretoria, South Africa; 2 Musculoskeletal Research Unit, School of Clinical Sciences, University of Bristol, Bristol, UK; 3 Department of Health Systems and Policy, University of Malawi, Zomba, Malawi; 4 College of Health Sciences, University of Zimbabwe-University of California, Avondale, Harare Zimbabwe; 5 San Francisco Collaborative Research Programme, University of Zimbabwe, Avondale, Harare Zimbabwe; 6 47 Newstead Road, Old Marlborough, Harare Zimbabwe

**Keywords:** Developing countries, Cervical cancer, HIV, Prevention

## Abstract

**Background:**

Over 85% of cervical cancer cases and deaths occur in developing countries. HIV-seropositive women are more likely to develop precancerous lesions that lead to cervical cancer than HIV-negative women. However, the literature on cervical cancer prevention in seropositive women in developing countries has not been reviewed. The aim of this study is to systematically review cervical cancer prevention modalities available for HIV-seropositive women in developing countries.

**Methods/design:**

This protocol was developed by following the Preferred Reporting Items for Systematic Reviews and Meta-Analyses Protocols (PRISMA-P) statement, and the systematic review will be reported in accordance with the PRISMA guidelines. Embase, MEDLINE, PubMed, CINAHL and Cochrane Library will be searched from inception up to date of final search, and additional studies will be located through citation and reference list tracking. Eligible studies will be randomised controlled trials, prospective and retrospective cohort studies, case-control and cross-sectional studies carried out in developing countries. Studies will be included if they are published in English and examine cervical cancer prevention modalities in HIV-seropositive women. Results will be summarised in tables and, where appropriate, combined using meta-analysis.

**Discussion:**

This review will address the gap in evidence by systematically reviewing the published literature on the different prevention modalities being used to prevent cervical cancer in HIV-seropositive women in developing countries. The findings may be used to inform evidence-based guidelines for prevention of cervical cancer in seropositive women as well as future research.

**Systematic review registration:**

PROSPERO CRD42017054678.

**Electronic supplementary material:**

The online version of this article (doi:10.1186/s13643-017-0484-9) contains supplementary material, which is available to authorized users.

## Background

Cervical cancer morbidity and mortality constitutes a growing burden in developing countries like Zimbabwe, Kenya, India, Botswana and South Africa; concern has shifted to how much can be done to prevent this public health challenge in all women with a lifetime risk approaching 1 in 20 in some developing settings [1]. A systematic review on the cervical cancer screening and prevention indicated that about 88% of all cervical cancer worldwide occurs in developing countries where there is very limited allocated resources to prevent and treat cervical cancer [1]. Research has shown that one has to be infected with human papilloma virus (HPV) to develop cervical cancer, but HPV alone does not fully explain cervical cancer epidemiology hence a number of cofactors associated [2, 3].

With the adverse of HIV in most of these developing countries especially those in sub-Saharan Africa, the burden of cervical cancer is increasing. HIV, which is a risk factor for cervical cancer, lowers women’s immune system, making them more susceptible to HPV infection [4–7]. Globally, 1 to 2% of HIV-negative women develop cervical intraepithelial neoplasia (CIN) stages 2 and 3 annually whilst HIV-positive women are at 10% more prone to developing CIN stages 2 and 3 [8]. The adverse of HIV/AIDS in most developing countries has resulted in high cervical cancer prevalence and because of this; cervical cancer has been classified as an AIDS-defining disease [5–7].

HIV-seropositive women have been found to be at higher risk of HPV infection due to their immune compromised status and that they are 2 to 12 times more likely to develop cervical precancerous lesions that lead to cervical cancer than HIV-negative women [5, 6]. In a case-control study in South Africa, HIV-seropositive women infected with HPV had a more than 40-fold higher risk of developing cervical squamous intraepithelial lesions compared to women who are both HIV and HPV negative [7].

Although cervical cancer screening in HIV-seropositive women has been found to reduce cervical cancer morbidity [8–10], challenges and constraints still exists in developing countries that make it difficult for cervical cancer screening to be available. Most developing countries do not have adequate or well-equipped laboratories, good quality control, qualified pathologists and cytotechnicians who are able to analyse and interpret laboratory specimen hence cervical cancer screening using HPV DNA, and Pap smear might not be available or it is expensive [10–12]. In cases where Pap smear or visual inspection with acetic acid (VIA) is available, it is mostly found in big urban clinics and hospitals and comes with a cost that many women cannot afford as well as being on waiting lists for months. 

Therefore, what is lacking in most developing countries is a systematic and organised population-based screening. This lack of organised and systematic population-based screening has resulted in cervical cancer screening among HIV-seropositive women to be poor, uncoordinated and sometimes undocumented. With research to test the efficiency of HPV vaccines on HIV-positive women currently underway, little is known on how effective it is in preventing cervical cancer in HIV-positive women [10]. The current WHO guidelines on the prevention of cervical cancer include cytology, VIA and HPV test. Besides the current research, most developing countries are yet to offer HPV vaccination, and this might not happen anytime soon because of lack of financial resources and technical expertise. Therefore, we aimed to review the available modalities used in the prevention of cervical cancer in HIV-seropositive women in developing countries so as to answer the following questions:

- Are cervical cancer prevention modalities being used for HIV-seropositive women different between countries?
- Have cervical cancer prevention modalities being used for HIV-seropositive women improved over time?
- Are cervical cancer prevention modalities being used for HIV-seropositive women effective in preventing cervical cancer?

## Methods/design

The development and reporting of this protocol was guided by the Preferred Reporting Items for Systematic Reviews and Meta-Analyses (PRISMA) Protocols (PRISMA-P) statement [13], and the systematic review will be carried out in accordance with the PRISMA guidelines [14].

### Protocol registration

This review protocol was registered with PROSPERO database (registration number: CRD42017054678) [15].

### Studies’ eligibility criteria

Studies will be included if

- The sample/population of interest are women
- Cervical cancer prevention methods for HIV-positive women (such as Pap smear, visual inspection with acetic acid, HPV DNA testing and HPV vaccination among others) are key outcomes
- HIV and cervical cancer prevention modalities are considered being independent and outcome variables, respectively
- Description, effect or impact of the prevention modality on HIV-seropositive women is an outcome
- Published in peer-reviewed journals
- Done in or for countries or regions considered to be developing countries by United Nations [16]
- They are randomised controlled trials and observational study designs—prospective cohorts, retrospective cohorts, case-control and cross-sectional
- Reported in English language
- Prospective cohort studies have a defined length of follow-up. Length of follow-up will be used to assess for the quality of the outcomes.

Studies will not be excluded based on length of follow-up. However, follow-up rates will be used to give scores to the quality of the outcomes. Follow-up rates of less than 60% are going to be considered as having limited validity especially when the reasons for loss are related to both exposure and outcome status [17]

In cases of studies done across countries, that are developed and developing, the team will extract results for developing countries from these where possible and may contact study authors if not available from paper. All studies will be included, and sample sizes will be used to assess quality and inform interpretation of findings. The reviewers’ assumptions are that studies with smaller samples might not provide additional value in terms of high quality evidence [18]. Reviews and studies looking at cervical cancer in general and those with unrepresentative samples will be excluded. Unrepresentative samples (looking at HIV-positive women, controls and sampling criteria) will be identified through performing non-parametric tests on geographical and demographical representation of the sample against that of the population.

### Search strategy

**Fig. 1:**
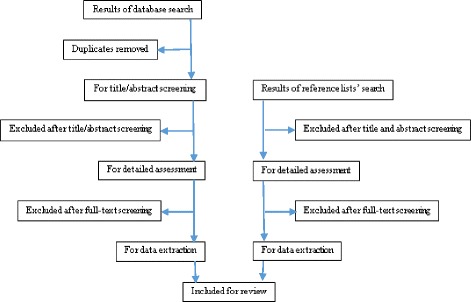
PRISMA review flow chart

The search system through the online databases will be based on the OVID search system. Key words will be used and will be supplemented by free-text terms (synonyms) of keywords to locate all the potential eligible articles. The search will be up to date of the final search. The following databases will be searched, PubMed (via the PubMed/MEDLINE interface using the “PICO” option), Cochrane (via The Cochrane Library using MeSH terms and qualifiers), CINAHL (via the EBSCO interface using key words), Embase and MEDLINE (via the OvidSP interface using key words (Table 1)). Truncation commands (root word) and proximity operators (words which will be within a chosen distance of each other) and Boolean logic operators (OR and AND) will be used as well (Table 2). To improve the efficiency of the final search, preliminary trials with search terms will be conducted. Citation and reference tracking will be used to search additional papers to add to the electronic database search, as shown in the PRISMA flow chart (Fig. 1).

**Table 1:** MEDLINE and Embase search strategy via OvidSP

Search terms
1. cervi* canc*.mp. [mp = title, abstract, full text, caption text]
2. cervi* neoplas*.mp. [mp = title, abstract, full text, caption text]
3. cervi* carcinom*.mp. [mp = title, abstract, full text, caption text]
4. cervi* dysplas*.mp. [mp = title, abstract, full text, caption text]
5. cervi* intraepithelial neoplas*.mp. [mp = title, abstract, full text, caption text]
6. prevent* or screen*.mp. [mp = title, abstract, full text, caption text]
7. pap smear*.mp. [mp = title, abstract, full text, caption text]
8. colposcopy.mp. [mp = title, abstract, full text, caption text]
9. hpv adj3 vaccin*.mp. [mp = title, abstract, full text, caption text]
10. HIV positive.mp. [mp = title, abstract, full text, caption text]
11. hiv seropositiv*.mp. [mp = title, abstract, full text, caption text]
12. hiv.mp. [mp = title, abstract, full text, caption text]
13. developing countr*.mp. [mp = title, abstract, full text, caption text]
14. underdeveloped countr*.mp. [mp = title, abstract, full text, caption text]
15. low income countr*.mp. [mp = title, abstract, full text, caption text]
16. low resource countr*.mp. [mp = title, abstract, full text, caption text]
17. low resource setting*.mp. [mp = title, abstract, full text, caption text]
18. developing countries.mp. [mp = title, abstract, full text, caption text]
19. 1 or 2 or 3 or 4 or 5
20. 6 or 7 or 8 or 9
21. 10 or 11 or 12
22. 13 or 14 or 15 or 16 or 17 or 18
23. 19 and 20 and 21 and 22

**Table 2:** Techniques to be used in the online databases search

Techniques	Description	Example
Free-text synonyms of keyword search	All known synonyms of the keyword in both British and US spellings	Cervical cancer synonyms: cervical carcinomas, cervix neoplasms, cervical intraepithelial neoplasia, cervix dysplasia etc.
Truncation commands	Using the root word to capture alternative word endings	Cervi* carcinom* searches for words such as cervical carcinoma, cervix carcinomas etc.
Proximity operators	Operators used will be Adj3 in OvidSP interface	hpv adj3 vaccin*
Boolean logic operators	‘OR’ and ‘AND’ will be the two commands to be used. ‘OR’ is used to locate articles with at least one of the search terms. ‘AND’ is to be used near the end of a search so as to combine results of different search concepts.	prevent* or screen* OR pap smear* OR colposcopy OR hpv adj3 vaccin* (prevent* or screen* OR pap smear* OR colposcopy OR hpv adj3 vaccin*) AND (HIV positive OR hiv seropositiv* OR hiv)

### Study selection

Merging of the results of the database searches will be done and two independently working researchers (from among WM, FM, SF and TC), will screen for abstracts. The full-text screening form (see Additional file 1) will be used to select potentially eligible papers. The papers will be double-screened and reasons for exclusions will be documented. Through discussions, disagreements and other issues pertaining to the screening process will be resolved among WM, AE, FM, SF and TC.

### Data extraction

Data will be double extracted by WM, FM, SF, TC, and discrepancies and disagreements will be resolved through discussions. The data extraction form (see Additional file 2) will be pretested/piloted on a few selected studies and will be adjusted accordingly for its appropriateness. The following data will be extracted from the included studies: publication year of the study, title of the study, study design, study setting (country/region), sample size, exposures and outcomes, all statistics such as descriptive, odds/risk ratios, logistic and linear regression and confounders included in the analysis will be extracted.

### Quality assessment

A modified version of the Newcastle-Ottawa Quality Assessment Scale [19], see Additional file 3, will be used to ascertain the quality of all included studies during data extraction. Quality of the included studies will be based on the following: study design used to measure cervical cancer prevention and or screening, focus of research and key findings (that is if study is describing prevention modalities or comparing two or more prevention modalities), representativeness of participants and length of follow-up. Studies included in the review will be categorised into three groups, RCTs, observational studies with a control group and observational studies without a control group.

For RCT studies, we will assess whether (1) randomization of participants is reported, (2) all participants who entered the study would have been accounted for in the analysis, (3) participants were analysed in the groups they were randomised to, (4) blinded outcome assessment was used, (5) power calculation information was provided, (6) baseline characteristics of study groups were balanced at the start of the study, and, in case were there was imbalance, adjusted for the imbalance was done in the analyses (Table 3).

**Table 3:** Randomised clinical trials quality assessment checklist

Assessment criteria	Studies fulfilling criteria	Studies not fulfilling criteria
Randomization of participants is reported		
All participants who entered the study would have been accounted for in the analysis		
Participants were analysed in the groups they were randomized to		
Blinded outcome assessment was used		
Power calculation information was provided		
Baseline characteristics of study groups were balanced or adjustment for the imbalance in analyses		

Observational studies with a control group will be assessed to see whether (1) participants, both groups, were stratified for the cervical cancer prevention or screening method under review, (2) if groups were not stratified for prevention and screening methods and the distribution was unbalanced, we will assess whether the outcomes were adjusted for (Table 4).

**Table 4:** Observational studies with a control group quality assessment checklist

Assessment criteria	Studies fulfilling criteria	Studies not fulfilling criteria	Studies not applicable
Assessment of participants’ on admission to study			
Assessment of prevention method under review			
Participants were stratified for the cervical cancer prevention or screening method under review			
Ascertainment of cervical cancer and HIV status, prospectively from participants through diagnosis, laboratory tests and blood tests			
Ascertainment of cervical cancer and HIV status, retrospectively from participants through diagnosis, laboratory tests and blood tests			
Complete follow-up—all subjects accounted for			
Subjects lost to follow up unlikely to introduce bias (≥75% follow-up or description provided of those lost			
If groups were not stratified for prevention and screening methods and the distribution was unbalanced, were outcomes adjusted for			

For observational studies without a control group, we will assess whether (1) the study population was a consecutive cohort of participants, (2) included participants have fulfilled predefined criteria, (3) study design (prospective or retrospective) information was given (Table 5).

**Table 5:** Observational studies without a control group quality assessment checklist

Assessment criteria	Studies fulfilling criteria	Studies not fulfilling criteria
Study population was a consecutive cohort of participants		
Included participants have fulfilled predefined criteria		
Study design information given		

For the outcome measures in all study groups, we will assess whether (1) a predefined outcome measure was defined and (2) any method or cervical cancer prevention or screening was used or information on its application was given (Table 6).

**Table 6:** Outcome measures’ quality assessment checklist

Methods of screening/prevention	Assessment criteria	Studies fulfilling criteria	Studies not fulfilling criteria
Pap smear	Clinical definition		
	Technical investigation		
	Definition of abnormal results		
Visual inspection with acetic acid	Clinical definition		
	Technical investigation		
	Definition of abnormal results		
HPV vaccination	Clinical definition		
	Technical investigation		
	Definition of abnormal results		
Other prevention/screening methods	Clinical definition		
	Technical investigation		
	Definition of abnormal results		

Screening of search results, quality examination and extraction of relevant data, will be carried out by two independently working researchers. Any discrepancies and disagreements that arise during the review study will be resolved through discussion. The average of the two reviewers will be the quality score for each study, where a range of zero (lowest quality) to five (highest quality) will be used. Studies will not be excluded based on quality rating but quality results will be included in the synthesis of the findings.

### Synthesis

Results of this review are going to be synthesised in two forms, that is, narrative synthesis and meta-analysis [20]. The narrative synthesis will summarise the results and characteristics of the included studies through the use of tables. Where sufficient consistency is found in the reporting of the methods and results across different studies, meta-analysis will be used to combine the numerical findings, e.g., odds/ratio ratios, daily-adjusted life years (DALY) of the effect or impact of the prevention modality on HIV-seropositive women so as to provide adequate estimates.

Random-effects aggregate data meta-analysis will be used to combine the findings from the different studies which will be included in the review. If included studies have sufficient details to extract data of participants’ ages or duration of HIV infection, then subgroup analysis to answer specific questions about different participants’ groups will be performed. Estimates during analysis will be presented in forest plots and tables. The meta-analysis will be run using the STATA Statistical package. Data entry will be done by the two independent reviewers so as to limit typing errors.

The meta-analysis will also be used for the review’s sensitivity analysis and assessment of bias. Judgement and risk of bias table will be used through a risk of bias tool specific for each included study. Through judgement, reviewers will assess the risk of bias as follows: ‘low risk’, ‘high risk’ or ‘unclear risk’ when there is lack of information on bias. Funnel plots of risk of bias will be created using RevMan software and *t* test applicable at probability of 95% will be performed for statistical significance. Heterogeneity between the analysed studies will be assessed using Higgins and Thompson’s *I* ^2^ statistic [21, 22]. An *I* ^2^ statistic of 0% indicates no observed heterogeneity and larger values indicate increasing heterogeneity, and this will be significant at *p* value less than or equal to 0.05.

### Reporting

This systematic review and its findings will be reported in accordance with the PRISMA statement.

## Conclusions

The evidence generated from this review will be used to address the gaps that might exist in the prevention and screening of cervical cancer in HIV-seropositive women as well as informing future research, cervical cancer policies and cervical cancer interventions. The strengths and limitations for this review will be considered, and the review findings will be discussed in the context of other reviews and evidence that are relevant.

## Additional files

Additional file 1: Full-text screening form. This form will be used for the screening of potentially eligible studies to be included in the review. (DOCX 15 kb)

Additional file 2: Data extraction form. This form will be used to extract relevant data such as exposure and outcome and statistics from the included studies. (DOCX 18 kb)

Additional file 3: Quality assessment form. This form will be used for grading the quality of studies included in the review. It is a modified/amended version of the Newcastle-Ottawa Quality Assessment Scale. (DOCX 21 kb)
