# Supplementary material for: Prevention of cervical cancer in HIV-seropositive women from developing countries: a systematic review protocol
Source: Syst Rev. 2017 Apr 24;6:91. doi: 10.1186/s13643-017-0484-9 (PMC5404686; doi:10.1186/s13643-017-0484-9)
Supplement: Supplementary file 1 — Full-text screening form. This form will be used for the screening of potentially eligible studies to be included in the review. (DOCX 15 kb) [file 13643_2017_484_MOESM1_ESM.docx]

**File 2**

Form 1 Full-text screening: **PREVENTION OF CERVICAL CANCER IN HIV SEROPOSITIVE WOMEN FROM DEVELOPING COUNTRIES: A SYSTEMATIC REVIEW**

| Reference details |  |  |  |  |  |  |  |  |  |  |  |  |
| --- | --- | --- | --- | --- | --- | --- | --- | --- | --- | --- | --- | --- |
| A1. Ref ID |  | | | | | | | | | | | |
| A2. 1^st^ Author (pub. year) |  | | | | | | | | | | | |
| A3. Title of article |  | | | | | | | | | | | |
| A4. Journal |  | | | | | | | | | | | |
| A5. Publication type | Paper |  | Abstract | | | | | | | | |  |
| A6. Assessor’s name | XX |  | XX |  | XX |  | XX |  | XX |  | XX |  |
| A7. Date |  | | | | | | | | | | | |

| Yes^1^ |  | No^2^ |  |
| --- | --- | --- | --- |

B. Study included in systematic review:

| Reason(s) for exclusion (if excluded): | Yes^1^ | No^2^ |
| --- | --- | --- |
| C1. Prevention methods for cervical cancer in HIV seropositive women not investigated. |  |  |
| C2. Unrepresentative sample |  |  |
| C3. Done in developed countries |  |  |
| C4. Review/editorial article |  |  |
| C5. Duplicate (Insert Ref ID of other study) |  |  |
|  | Ref ID: |  |
| C6. Other please specify: | | |
